# Elevation of pro-inflammatory cytokine levels following anti-resorptive drug treatment is required for osteonecrosis development in infectious osteomyelitis

**DOI:** 10.1038/srep46322

**Published:** 2017-04-07

**Authors:** Mayu Morita, Ryotaro Iwasaki, Yuiko Sato, Tami Kobayashi, Ryuichi Watanabe, Takatsugu Oike, Satoshi Nakamura, Yosuke Keneko, Kana Miyamoto, Kazuyuki Ishihara, Yoichiro Iwakura, Ken Ishii, Morio Matsumoto, Masaya Nakamura, Hiromasa Kawana, Taneaki Nakagawa, Takeshi Miyamoto

**Affiliations:** 1 Division of Oral and Maxillofacial Surgery, Department of Dentistry and Oral Surgery Keio University School of Medicine, 35 Shinano-machi, Shinjuku-ku, Tokyo 160-8582, Japan; 2 Department of Orthopedic Surgery Keio University School of Medicine, 35 Shinano-machi, Shinjuku-ku, Tokyo 160-8582, Japan; 3 Department of Advanced Therapy for Musculoskeletal Disorders Keio University School of Medicine, 35 Shinano-machi, Shinjuku-ku, Tokyo 160-8582, Japan; 4 Department of Musculoskeletal Reconstruction and Regeneration Surgery, Keio University School of Medicine, 35 Shinano-machi, Shinjuku-ku, Tokyo 160-8582, Japan; 5 Department of Microbiology, Tokyo Dental College, 2-9-18 Misakicho Ciyoda-ku, Tokyo, 101-0061, Japan; 6 Division of Experimental Animal Immunology, Center for Animal Disease Models, Research Institute for Biomedical Sciences, Tokyo University of Science, 2641 Yamazaki, Noda-shi, Chiba 278-8510, Japan

## Abstract

Various conditions, including bacterial infection, can promote osteonecrosis. For example, following invasive dental therapy with anti-bone resorptive agents, some patients develop osteonecrosis in the jaw; however, pathological mechanisms underlying these outcomes remain unknown. Here, we show that administration of anti-resorptive agents such as the bisphosphonate alendronate accelerates osteonecrosis promoted by infectious osteomyelitis. Potent suppression of bone turnover by these types of agents is considered critical for osteonecrosis development; however, using mouse models we found that acceleration of bone turnover by teriparatide injection did not prevent osteonecrosis but rather converted osteoclast progenitors to macrophages expressing inflammatory cytokines, which were required for osteonecrosis development. In fact, we demonstrate that TNFα-, IL-1α/β- or IL-6-deficient mice as well as wild-type mice administered a TNFα-inhibitor were significantly resistant to development of osteonecrosis accompanying infectious myelitis, even under bisphosphonate treatment. Our data provide new insight into mechanisms underlying osteonecrosis and suggest new ways to prevent it.

Osteonecrosis is characterized by osteocyte death, as marked by the appearance of empty lacunae in cortical bone^1^,^2^, and develops in response to trauma, radiation, bacterial infection or ischemia^3^,^4^,^5^,^6^,^7^,^8^,^9^. Most osteonecrosis is intractable and often causes bone fragility fractures or bone deformities^10^,^11^,^12^. Osteonecrosis of the jaw (ONJ) was initially reported in patients who had undergone invasive dental treatment such as tooth extraction or implantation and had been administered long term the bisphosphonate alendronate, which potently inhibits bone resorption and suppresses bone turnover^13^,^14^. Such osteonecrosis was initially termed bisphosphonate-related ONJ (BRONJ)^15^. ONJ was subsequently seen in patients treated with desnosumab, an antibody targeting receptor activator of nuclear factor kappa B ligand (RANKL), a different anti-resorptive agent. As a result, ONJ seen in patients treated with anti-resorptive agents in general is now called ARONJ (anti-resorptive agent-related ONJ)^16^. To date, ARONJ is reported in patients with either osteoporosis or metastatic bone tumors who were administered strong anti-bone resorbing agents, including but not limited to bisphosphonates and denosumab^16^. ARONJ is intractable and causes severe deficits in quality of life^17^; thus preventing osteonecrosis is critical for maintenance of activity of daily living in ARONJ patients.

Given these outcomes and that metastatic bone cancer patients administered particularly high doses of anti-resorbing agents frequently exhibit ARONJ, investigators have concluded that either long-term usage or high doses of these drugs promotes a condition known as severely suppressed bone turnover (SSBT), which associated with osteonecrosis development^18^,^19^. Interestingly, treatment with teriparatide, a parathyroid hormone (PTH) peptide that activates bone turnover, is reportedly effective in treating ARONJ patients^20^,^21^.

In addition to SSBT, local infection is thought to promote ARONJ^8^, since jaws are near the oral cavity where >10^11^ bacteria/cm^3^ exist and may be released following invasive dental treatment. ARONJ is also seen in individuals with poor oral hygiene or diabetes myelitis or rheumatoid arthritis (RA) patients, who are often at high risk for infection^8^,^12^. At present, a relationship between the use of anti-resorbing agents and infection is not known.

To date, several ONJ animal models have been reported^22^,^23^,^24^,^25^,^26^. For example, immune-deficient beige nu/nu Xid (III) mice exhibit ARONJ-like phenotypes following tooth extraction and treatment with the bisphosphonate zoledronate and the steroid dexamethasone^27^. ONJ is also reportedly seen in bisphosphonate-treated rats^23^, dogs^28^ and other species^29^. In contrast, ONJ is not seen in C57/BL6 wild-type mice undergoing tooth extraction and zoledronate treatment, although mice exhibit delayed wound healing^30^. The activity of regulatory T cells (Tregs)^27^ or γδ T cells^31^ reportedly promotes ARONJ development, although it is unclear how these activities promote osteonecrosis development.

Here, we hypothesized that ARONJ develops from a combination of infectious myelitis and treatment of anti-resorptive agents. Using mice, we show that alendronate treatment exacerbates osteonecrosis development in femur in cases of infectious osteomyelitis. Accelerating bone turnover by teriparatide administration did not prevent osteonecrosis development in this model. Following alendronate treatment of mice with infectious osteomyelitis, osteocytes underwent apoptosis. Moreover, in the presence of alendronate, osteoclast progenitor cells were converted to TNFα-, IL-6- and IL-1β-expressing cells *in vitro*, and blocking TNFα, either by gene targeting or treatment with the TNFα-inhibitor etanercept, significantly inhibited osteonecrosis development, even in alendronate-treated mice with infectious osteomyelitis mice. IL-6- or IL-1α/β-deficient mice were also significantly resistant to osteonecrosis development in this model. Our data provide insight into osteonecrosis pathogenesis and suggest therapeutic targets potentially useful to antagonize ONJ, even in the presence of anti-resorptive therapy.

## Results

### Anti-bone resorbing agents accelerate osteonecrosis development in the presence of infectious osteomyelitis

**Figure 1:**
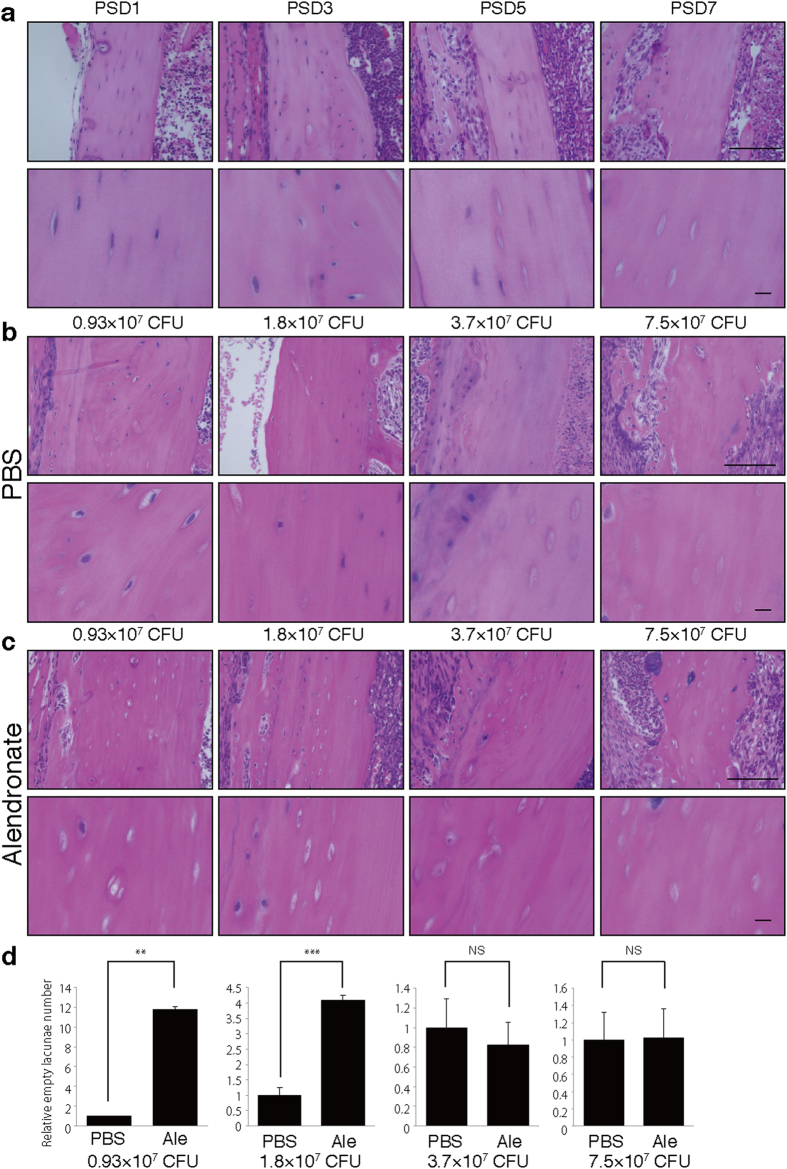
Alendronate treatment accelerates osteonecrosis development. Wild-type mice were administered alendronate (Ale) or vehicle (PBS) for two weeks. Then, infectious osteomyelitis was established by direct surgical application of 7.5 × 10^7^ (**a**) or indicated (**b**–**d**) colony-forming units (CFU) of *Streptococcus aureus* (SA) to femurs. After indicated (**a**) or seven (**b**–**d**) days post surgery (post surgery day, PSD), sections of cortical bone were stained with HE, observed microscopically **(a-c)**, and the number of empty versus whole lacunae was scored. Data represents relative proportion of empty versus whole lacunae in cortical bone ± SD (*n* = 4). Scale bars = 100 μm (upper) and 10 μm (lower panels). **P < 0.01; ***P < 0.001; NS, not significant. Representative data of at least two independent experiments are shown.

To assess effects of anti-resorptive bisphosphonates on osteonecrosis, we created a murine model of osteonecrosis by first pre-treating wild-type mice with alendronate (Ale) by injection and then surgically infecting the left femurs with *Streptococcus aureus* (SA) (Fig. 1). Left femurs were sham-operated and served as non-infected controls. Osteonecrosis, as marked by the appearance of empty lacunae in cortical bone, was not seen in the sham-operated femur of alendronate-treated mice (Alendronate/PBS) (Fig. S1) but appeared in mice treated with a combination of SA and alendronate in a time- and SA titer-dependent manner (Fig. S1a–d). This observation suggests that administration of alendronate alone is not sufficient for osteonecrosis development. Higher SA titers (>3.7 × 10^7^ CFU) promoted osteonecrosis development in the absence of alendronate (PBS group) (Fig. 1b and d). However, osteonecrosis developed even at lower SA titers (<1.8 × 10^7^ CFU) in alendronate-treated mice (Fig. 1c and d), suggesting that alendronate accelerates osteonecrosis development.

Similar to outcomes seen in alendronate-treated mice, osteonecrosis developed in mice administered anti-RANKL antibody, a different anti-resorptive agent (Fig. S2), suggesting that in the presence of osteomyelitis, treatment with any anti-bone resorbing agent promotes osteonecrosis development.

Overall, osteonecrosis was induced in osteocytes in ratio indicated in Fig. S1 in 100% of mice treated with a combination of SA and anti-bone resorptive agents.

### Osteocytes in alendronate-treated mice with infectious osteomyelitis undergo apoptosis

Treatment of patients with teriparatide, a bioactive form of PTH, reportedly antagonizes SSBT^20^,^21^. Thus, we administered teriparatide to alendronate-treated osteomyelitis model mice by subcutaneous injection at the time of SA infection and then twice weekly (Fig. S3). Osteonecrosis development was, however, not blocked by teriparatide treatment (Ale + PTH) but rather developed at levels comparable to those seen in mice not treated with teriparatide (Ale) (Fig. S3), suggesting that SSBT in this context does not promote osteonecrosis development. Moreover, bone turnover as evaluated by osteoid production was inhibited by alendronate treatment but reversed by PTH (Fig. S4a). In contrast, proliferation of bone lining osteoblastic cells, as analyzed by staining for proliferating cell nuclear antigen (PCNA), was inhibited by alendronate and not fully rescued by PTH treatment (Fig. S4b). Teriparatide administration did not promote up-regulation of bone-turnover markers in sera in patients treated with alendronate^32^. However, administration of alendronate alone did not promote osteonecrosis development, strongly suggesting that SSBT is not the primary cause of osteonecrosis.

**Figure 2:**
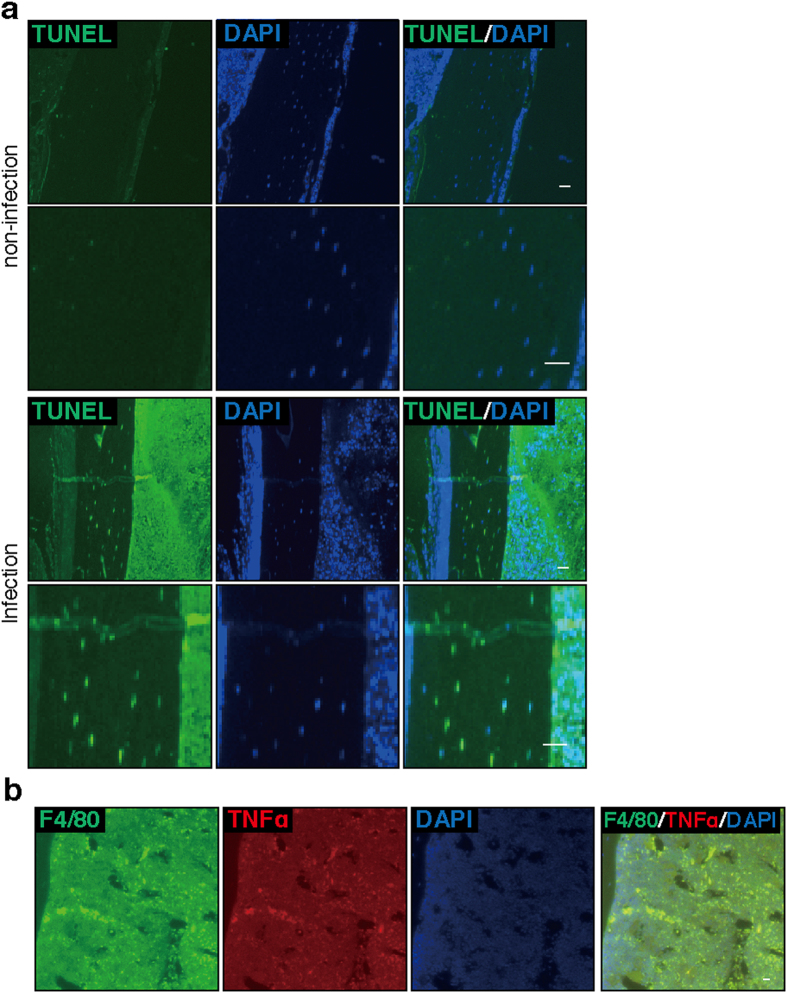
Alendronate treatment promotes osteocyte apoptosis and stimulates TNFα expression in macrophages. Wild-type mice were administered alendronate for two weeks. Then, osteomyelitis (infection) was established in left femurs, as in Fig. 1. Right femurs were sham-operated and served as controls (non-infection). Seven days later, bone sections were prepared and labeled with Biotin-dUTP using terminal deoxynucleotidyl trans (TdT), followed by Avidin-DTAF as TUNEL staining (TUNEL) to identify apoptotic cells (**a**). Sections were doubly-stained with Alexa488-conjugated rat anti-mouse F4/80 and goat anti-mouse TNFα, followed by Alexa488-conjugated donkey anti-rat Ig’ and Alexa568-conjugated donkey anti-goat Ig’. (**b**). Nuclei were stained with DAPI. Sections were observed under a fluorescence microscope. Bar = 10 μm. Representative data of at least two independent experiments are shown.

Nonetheless, we found that osteonecrosis developed on the SA-infected but not the control, uninfected side of mice injected with alendronate (Fig. S5), suggesting that disease emerges from local rather than systemic factors in alendronate-administered, SA-infected mice. Furthermore, we observed apoptosis, as detected by a TUNEL assay, in osteocytes of infected and alendronate-treated mice (Fig. 2a). TNFα is a strong inducer of apoptosis^33^. In accordance, immunofluorescence analysis revealed TNFα expression in F4/80-positive macrophages in osteomyelitis mice treated with alendronate (Fig. 2b), suggesting an association of alendronate treatment with TNFα expression. Thus, we focused on TNFα rather than SSBT as a potential underlying cause of osteonecrosis seen in the presence of anti-resorptive agents.

### Infection in the presence of alendronate treatment converts osteoclast progenitors to TNFα-expressing cells

**Figure 3:**
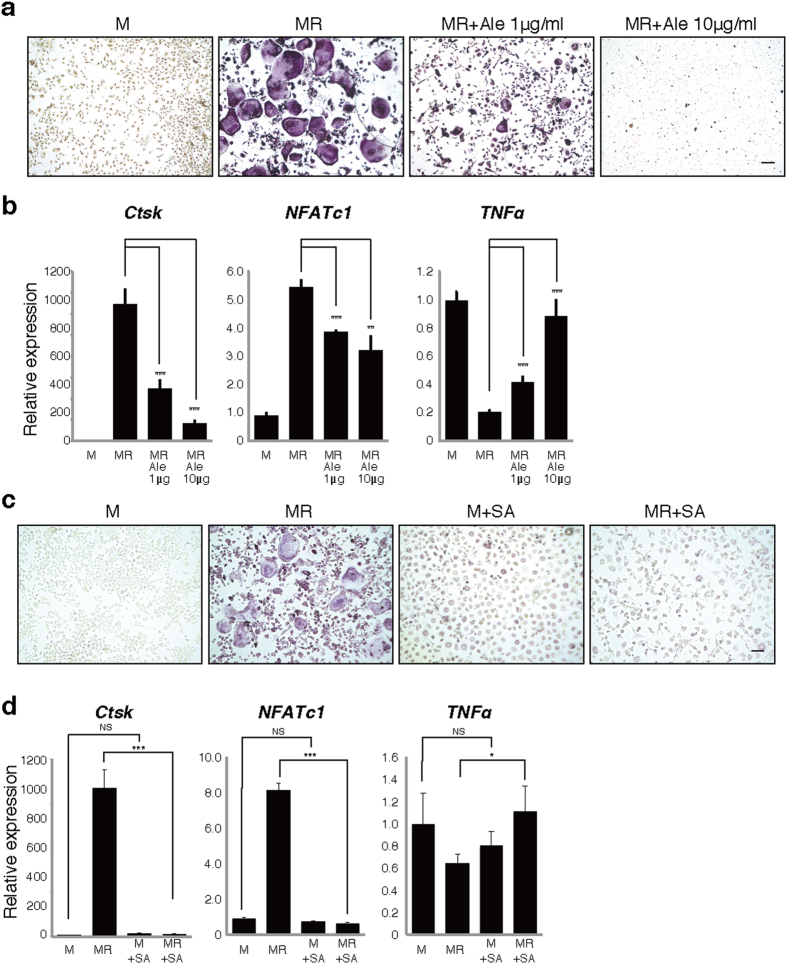
Alendronate treatment or *Streptococcus aureus* infection increases TNFα expression and inhibits osteoclastogenesis. Osteoclast progenitors were isolated from wild-type mice and cultured in the presence or absence of M-CSF (M) and RANKL (R) with or without 1 or 10 μg/ml alendronate (Ale) (**a**,**b**) or *Streptococcus aureus* lysate (SA) (**c**,**d**). Osteoclast formation was evaluated by TRAP staining (**a**,**c**), or realtime PCR to analyze expression of *Cathepsin K (Ctsk) and NFATc1* (**b**,**d**)*. TNFα* expression was also analyzed by realtime PCR(**b**,**d**). Scale bar = 100 μm. Data represent mean *Ctsk, NFATc1* or *TNFα* expression relative to *β-actin* ± SD (*n* = 3). *P < 0.05; **P < 0.01; ***P < 0.001; NS, not significant. Representative data of at least two independent experiments are shown.

To determine potential effects of anti-resorptive agents on osteoclast progenitors, we isolated M-CSF-dependent bone marrow macrophages (BMMs) as osteoclast progenitors and then cultured with M-CSF (M) and RANKL (R) in the presence or absence of various alendronate concentrations (Fig. 3). Interestingly, TNFα expression in BMMs was significantly downregulated by RANKL along with osteoclast differentiation, as indicated by induction of *Cathepsin K (Ctsk*) and *nuclear factor of activated T cells 1 (NFAtc1*), but was significantly stimulated by alendronate in a dose-dependent manner inversely correlated with inhibition of osteoclastogenesis (Fig. 3a and b).

Bacterial infection also strongly induces TNFα expression^34^. Indeed, TNFα expression was significantly upregulated by treatment of cultured BMMs with SA lysate even in the presence of RANKL (Fig. 3c and d).

Similar to SA, we also observed development of osteonecrosis following of development of infectious osteomyelitis by infection of femurs of alendronate-treated mice with *Porphyromonas gingivaris* (PG), a periodontal bacteria (Fig. S6a and b). *In vitro*, osteoclastogenesis was significantly inhibited while TNFα expression was significantly upregulated by PG treatment in osteoclast progenitor cells (Fig. S6c and d), as seen in SA-treated cells. These results suggest combined infection plus treatment with anti-resorptive agents promotes TNFα expression in osteoclast progenitors, and anti-resorptive agents convert the osteoclast progenitor cells into TNFα-expressing macrophages even in the presence of RANKL.

### TNFα is required for osteonecrosis in osteomyelitis mice administered alendronate

**Figure 4:**
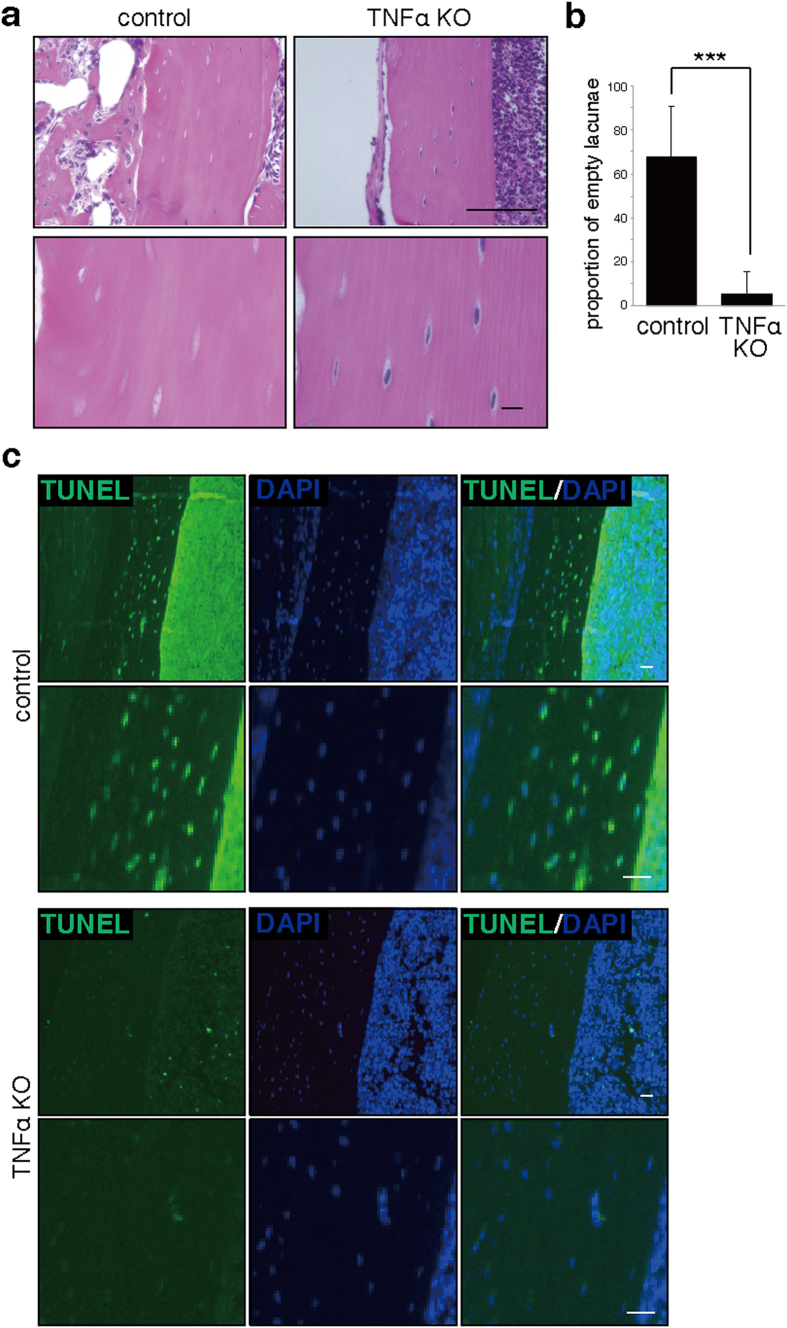
TNFα is required for osteocyte apoptosis accompanying osteonecrosis. Wild-type (control) or TNFα-deficient (TNFα KO) mice were administered alendronate for two weeks. Infectious osteomyelitis was then established by *Streptococcus aureus* infection of left femurs of control (wild-type) or TNFα KO mice. One week later, cortical bone sections were prepared and stained with HE (**a**) or TUNEL (**c**), and the proportion of empty to whole lacunae in cortical bone was determined (**b**). DAPI served as a nuclear stain (**c**). Data shows the mean percentage (%) of empty versus whole lacunae ± SD (*n* = 4, ***P < 0.001). Scale bars = 100 (upper in (**a**) or 10 μm (lower panels in (**a**), and all panels in (**c**). Representative data of at least two independent experiments are shown.

To determine whether TNFα is required for osteonecrosis development in our model system, we induced infectious osteomyelitis using a comparable experimental protocol in TNFα-deficient (TNFα KO) mice administered alendronate (Fig. 4). We observed that osteonecrosis promoted by infection was significantly blocked in TNFα-deficient mice (Fig. 4a and b). Likewise, the number of either apoptotic TUNEL-positive or necrotic ssDNA-positive osteocytes decreased significantly in TNFα-deficient mice (Fig. 4c and Fig. S7), suggesting that TNFα is required for apoptosis induction.

In experiments with wild-type mice, we also observed significant upregulation of the pro-inflammatory cytokines *IL-1β, IL-6, IL-17a* and *IL-17f* in infectious osteomyelitis tissue relative to control femurs in SA-infected, alendronate-treated (SA+, Ale+) versus infected but non-treated (SA+, Ale−) controls (Fig. S8). These results suggest that inflammatory cytokine expression by infection was further stimulated by alendronate treatment. Interestingly, osteonecrosis induced by infectious osteomyelitis plus alendronate administration was significantly blocked in either IL-1α/β (IL-1 KO)- or IL-6-deficient (IL-6 KO) mice, and was weakly attenuated in IL-17A/F-deficient (IL-17 KO) mice (Fig. S9).

### Administration of TNFα antagonists or vitamin D analogues antagonizes osteonecrosis in infected mice treated with anti-resorptive agents

**Figure 5:**
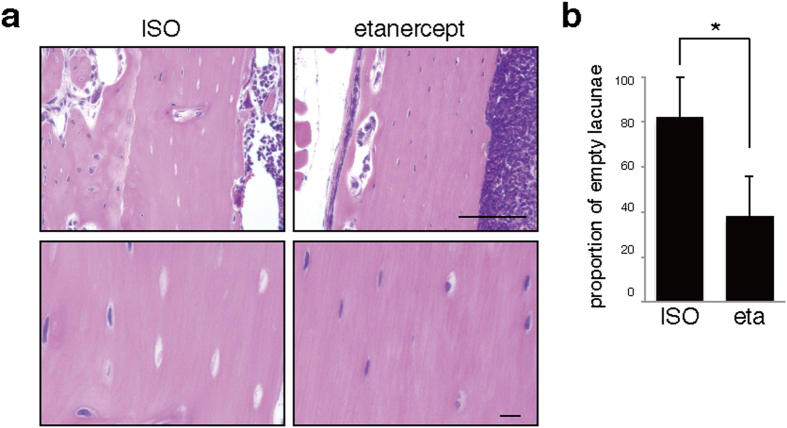
Blocking TNFα significantly antagonizes osteonecrosis development. Wild-type mice were administered alendronate for two weeks. Then, infectious osteomyelitis was established by *Streptococcus aureus* infection of left femurs. The TNFα inhibitor etanercept (eta) or ISO type control (ISO) was subcutaneously injected one week before surgery and then subsequently twice a week. Seven days after surgery, cortical bone sections of left femurs were prepared and stained with HE **(a)**, and the proportion of empty versus whole lacunae was calculated (**b**). Scale bars = 100 (upper) or 10 μm (lower panels). Data shows the mean percentage (%) of empty versus whole lacunae ± SD (*n* = 4, *P < 0.05). Representative data of at least two independent experiments are shown.

To develop potential treatments against osteonecrosis brought on by infectious osteomyelitis and treatment with anti-resorptive agents, we administered etanercept, a TNFα-inhibitor, subcutaneously one week before surgery and subsequently twice a week to alendronate-treated model mice infected in the left femur with SA (Fig. 5). Etanercept treatment significantly blocked osteonecrosis development in the infected femur (Fig. 5).

Active vitamin D, 1,25(OH)_2_D_3_ (VD3), or its analogue ED71, is often administered with anti-resorptive agents to osteoporosis patients^35^,^36^. We observed that administration of either VD3 or ED71 two weeks before surgery and subsequently twice weekly significantly blocked osteonecrosis in alendronate-treated mice with infectious osteomyelitis (Fig. S10). These studies suggest overall that either blocking TNFα or treatment with vitamin D analogues can prevent osteonecrosis development due to infection in mice treated with anti-resorptive agents.

Finally, we asked ectopic bone formation, which frequently occurs in ARONJ patients, resulting in facial deformities, a serious concern for patients^17^. We detected ectopic bone formation beneath the periosteum in mice treated systemically with alendronate on the side exhibiting infectious osteomyelitis but not on the control side (Fig. S11a). We observed similar outcomes following anti-RANKL treatment in the presence of SA infection or in alendronate-treated mice infected with PG (Fig. S11b and c). Our findings in mice are likely consistent with clinical observations, as ectopic bone formation in ARONJ patients is limited to the jaw, even in the presence of anti-resorptive treatment^37^,^38^,^39^. Interestingly, in our model of infectious osteomyelitis in the presence of alendronate, ectopic bone formation under the periosteum was significantly blocked by targeting of *TNFα*- or *IL-1α/β* or by treatment with a TNFα inhibitor (Fig. S11d and e). By contrast, ectopic bone formation was not blocked by treatment with PTH or vitamin D analogues (Fig. S11f and g).

## Discussion

**Figure 6:**
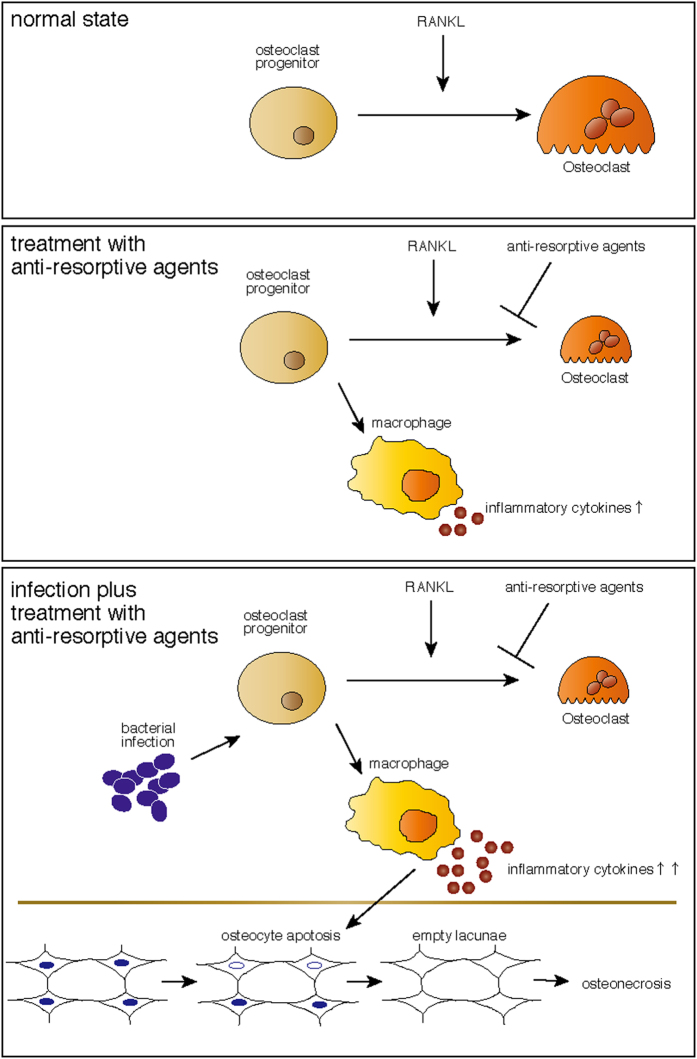
A schematic model for osteonecrosis development by osteomyelitis in the presence of administration of anti-resorptive agents. (upper) In the normal state, osteoclast progenitors differentiate into osteoclasts by RANKL exposure. (middle) Treatment with anti-resorptive agents converts osteoclast progenitors to inflammatory cytokine-expressing macrophages. (lower) Bacterial infection further promotes inflammatory cytokine expression, which in turn promotes osteocyte apoptosis, leading to osteonecrosis.

Treatment with anti-resorptive agents may promote ARONJ, and there is debate over whether these types of drugs should be withdrawn to prevent ARONJ prior to invasive oral treatment, despite fracture risk. In this study we show that alendronate treatment significantly exacerbates infectious osteomyelitis-induced osteonecrosis development. We found that osteonecrosis development was not blocked by teriparatide, which promotes bone turnover, suggesting that low bone turnover does not underlie osteonecrosis. Instead, we show that osteonecrosis development in infectious osteomyelitis mice administered alendronate was significantly blocked by gene targeting of either *TNFα*-, *IL-6* or *IL-1α/β*, or by treatment with a TNFα inhibitor. Moreover we observed that osteoclast progenitors were converted to inflammatory cytokine-expressing cells following alendronate treatment, even in the presence of RANKL. Thus, pro-inflammatory cytokines may represent therapeutic targets to prevent osteonecrosis induced by infectious osteomyelitis in patients treated with anti-resorptive therapy (Fig. 6).

ARONJ development in patients is limited to jaws, even in SSBT conditions promoted by treatment with anti-resorptive agents. Indeed, in our model, alendronate treatment alone did not promote osteonecrosis development, and osteonecrosis occurred in infectious osteomyelitis but not on the non-infectious control side in mice treated with alendronate, suggesting that local inflammation underlies osteonecrosis. Indeed, systemic inflammation is seen in RA patients, who are frequently treated with anti-resorptive agents to antagonize secondary osteoporosis. However, not all RA patients develop ARONJ, suggesting that strong local inflammation induced by infection plus potential conversion of osteoclast progenitors to inflammatory macrophages is a primary contributor to osteonecrosis.

We also found that although either VD3 or ED71 could prevent infectious myelitis from developing into osteonecrosis in the presence of alendronate treatment (Fig. S10), neither agent inhibited inflammatory cytokine expression by either macrophages or osteoclasts (Fig. S12). These results suggest that vitamin D or vitamin D analogues prevent osteonecrosis development via cells other than macrophages or osteoclasts or by mechanisms different from those that regulate inflammation. Further studies are needed to determine how VD3 and ED71 block osteonecrosis development.

To date, several ARONJ models have been established in mice. Osteonecrosis was reportedly seen following administration of the bisphosphonate zoledronate, following tooth extraction in wild-type mice^27^,^40^,^41^,^42^,^43^,^44^. These methods are considered more straightforward techniques to investigate ARONJ development compared with our models and thus represent a limitation of our study. Meanwhile, over 5–15 weeks of observation, osteonecrosis development in these models occurred in 0–40% of osteocytes^27^,^40^,^41^,^42^,^43^,^44^ in approximately 10% of wild-type mice^26^; however, our model promotes osteonecrosis development in three weeks in 100% of wild-type mice, and 60–80% of lacunae observed in the model were empty. This enabled us to analyze effects of inflammatory cytokines on osteonecrosis development. Infectious myelitis of the jaw is considered a cause of ARONJ development in humans who have undergone invasive dental treatment and are being treated with bisphosphonates^8^,^45^. Thus models combining infectious myelitis with bisphosphonate administration are useful to understand mechanisms underlying ARONJ development. Further studies are needed to fully understand pathological mechanisms underlying ARONJ development.

Our study suggests that it is critical to control inflammation in cases of invasive dental treatment in patients administered anti-resorptive agents to prevent ARONJ and ectopic bone formation. Anti-TNFα drugs or reagents that block IL-6 or IL-1 receptor agonists now clinically available may serve as useful therapies in this context.

## Methods

### Mice

Wild-type mice were purchased from Sankyo Labo Service (Tokyo, Japan). TNFα-deficient mice were purchased from The Jackson Laboratory (Bar Harbor, ME, USA). IL-1α−/− IL-1β−/−, IL-17-, and IL-6-deficient mice were prepared as described^1^,^2^,^3^. Animals were maintained under specific pathogen-free conditions in animal facilities certified by the Keio University Institutional Animal Care and Use Committee, and animal protocols were approved by that committee. Animals were housed up to 5 mice per cage and kept on a 12 h light/dark cycle. Water and food was available ad libitum. All animal studies were performed in accordance with the Guidelines of the Keio University animal care committee.

### Immunofluorescence

Surgical sections of bone tissue were stained using a MEBSTAIN Apoptosis TUNEL Kit Direct (MEDICAL & BIOLOGICAL LABORATORIES CO., LTD., Nagoya, Japan), or stained with anti-single standard DNA (ssDNA) Rabbit IgG (#18731 1:50; IBL, Gunma, Japan) followed by Alexa488-conjugated goat anti-rabbit Ig’ (#A-11034 1:200; Invitrogen, Carlsbad, CA). Sections were also stained with Alexa Fluor 488-conjugated rat anti-mouse F4/80 (#B116640 1:100 BioLegend, San Diego, CA, USA) and goat anti-mouse TNFα (#K2911 1:100 Santa Cruz Biotechnology, Inc., Santa Cruz, CA, USA), followed by Alexa488-conjugated donkey anti-rat Ig’ (#A-21208 1:200; Invitrogen, Carlsbad, CA) and Alexa568-conjugated donkey anti-goat Ig’ (#A-11057 1:200; Invitrogen, Carlsbad, CA). Sections were also stained with purified mouse anti-PCNA (#610664 1:100; BD Transduction Laboratories, Franklin Lakes, New Jersey) followed by Alexa488-conjugated goat anti-mouse Ig’ (#A-11029 1:200; Invitrogen, Carlsbad, CA). DAPI (#D1306 1:750; Wako Pure Chemicals Industries, Osaka, Japan) was used as a nuclear stain.

### *In vitro* osteoclast formation

Bone marrow cells isolated from wild-type mouse femurs and tibias were cultured 72 h in MEM (Sigma-Aldrich Co.) containing 10% (vol/vol) heat-inactivated FBS (JRH Biosciences) and GlutaMax (Invitrogen Corp.) supplemented with M-CSF (50 ng/mL, Kyowa Hakko Kirin Co.). Subsequently, adherent cells were collected and cultured in 96-well plates (1 × 105 cells per well) under indicated conditions containing M-CSF (50 ng/mL) and recombinant soluble RANKL (25 ng/mL, PeproTech Ltd.) with or without a SA or PG lysate. Lysates were prepared using RIPA buffer (1% Triton X-100, 1% sodium deoxycholate, 0.1% SDS, 150 mM NaCl, 5 mM EDTA, 1 mM dithiothreitol, 10 mM Tris-HCl, pH7.5) supplemented with a protease inhibitor cocktail (Sigma-Aldrich Co.) and MG-132 (EMD Millipore Corporation). Medium was changed every 2 days. Osteoclastogenesis was evaluated by TRAP staining, as described^4^,^5^.

### Quantitative PCR

Total RNAs were isolated from bone marrow cultures using TRIzol reagent (Invitrogen Corp.), and cDNA synthesis was performed using oligo(dT) primers and reverse transcriptase (Wako Pure Chemicals Industries). Quantitative PCR was performed using SYBR Premix ExTaq II reagent and a DICE Thermal cycler (Takara Bio Inc.), according to the manufacturer’s instructions. *β-actin (Actb*) expression served as an internal control. Primers for realtime PCR were as follows.

*β-actin*-forward: 5′-TGAGAGGGAAATCGTGCGTGAC-3′

*β-actin*-reverse: 5′-AAGAAGGAAGGCTGGAAAAGAG-3′

*Ctsk*-forward: 5′-ACGGAGGCATTGACTCTGAAGATG-3′

*Ctsk*-reverse: 5′-GGAAGCACCAACGAGAGGAGAAAT-3′

*NFATc1*-forward: 5′-CAAGTCTCACCACAGGGCTCACTA-3′

*NFATc1*-reverse: 5′-GCGTGAGAGGTTCATTCTCCAAGT-3′

*TNFα*-forward: 5′- AAGCCTGTAGCCCACGTCGT-3′

*TNFα*-reverse: 5′-GGCACCACTAGTTGGTTGTCTTTG -3′

*IL-1β*-forward: 5′-AAGTTGACGGACCCCAAAAGAT-3′

*IL-1β*-reverse: 5′-AGCTCTTGTTGATGTGCTGCTG-3′

*IL-6*-forward: 5′-GTCCTTAGCCACTCCTTCTG-3′

*IL-6*-reverse: 5′-CAAAGCCAGAGTCCTTCAGAG-3′

*IL-17a*-forward: 5′-TGTGAAGGTCAACCTCAAAGTCT-3′

*IL-17a*-reverse: 5′-CCCAGATCACAGAGGGATATCTA-3′

*IL-17f*-forward: 5′-TACTTCCTGAGGGAAGAAGCAG-3′

*IL-17f*-reverse: 5′-GCAAGTCCCAACATCAACAGTA-3′

### Statistical analysis

Results are expressed as means ± s.d. Statistical significance of differences between groups was evaluated using Student’s *t-*test (*P < 0.05; **P** < **0.01; ***P** < **0.001; NS, not significant, throughout the paper).

## Additional Information

**How to cite this article:** Morita, M. *et al*. Elevation of pro-inflammatory cytokine levels following anti-resorptive drug treatment is required for osteonecrosis development in infectious osteomyelitis. *Sci. Rep.* **7**, 46322; doi: 10.1038/srep46322 (2017).

**Publisher's note:** Springer Nature remains neutral with regard to jurisdictional claims in published maps and institutional affiliations.

## Supplementary Material

Supplementary Information
