# Supplementary material for: Elevation of pro-inflammatory cytokine levels following anti-resorptive drug treatment is required for osteonecrosis development in infectious osteomyelitis
Source: Sci Rep. 2017 Apr 7;7:46322. doi: 10.1038/srep46322 (PMC5384218; doi:10.1038/srep46322)
Supplement: Supplementary Information [file srep46322-s1.pdf]

Elevation of pro-inflammatory cytokine levels following anti-resorptive drug treatment is required for osteonecrosis development in infectious osteomyelitis

Mayu Morita[1], Ryotaro Iwasaki[1], Yuiko Sato[2,3], Tami Kobayashi[2,4], Ryuichi Watanabe[2], Takatsugu Oike[2], Satoshi Nakamura[2], Yosuke Keneko[2], Kana Miyamoto[2], Kazuyuki Ishihara[5], Yoichiro Iwakura[6], Ken Ishii[2], Morio Matsumoto[2], Masaya Nakamura[2], Hiromasa Kawana[1], Taneaki Nakagawa[1] and Takeshi Miyamoto[2, 3]

[1]Division of Oral and Maxillofacial Surgery, Department of Dentistry and Oral Surgery, [2]Department of Orthopedic Surgery, [3]Department of Advanced Therapy for Musculoskeletal Disorders, [4]Department of Musculoskeletal Reconstruction and Regeneration Surgery, Keio University School of Medicine, 35 Shinano-machi, Shinjuku-ku, Tokyo 160-8582, Japan; [5]Department of Microbiology, Tokyo Dental College, 2-9-18 Misakicho Ciyoda-ku, Tokyo, 101-0061, Japan; [6]Division of Experimental Animal Immunology, Center for Animal Disease Models, Research Institute for Biomedical Sciences, Tokyo University of Science, 2641 Yamazaki, Noda-shi, Chiba 278-8510, Japan

Supplementary Figure Legends

Figure S1 Alendronate administration alone does not promote osteonecrosis, as determined by formation of empty lacunae.

Wild-type mice were administered alendronate for two weeks, and then either vehicle (PBS) or *Streptococcus aureus* (SA) was surgically injected into left femurs. Seven days after surgery, cortical bone sections were prepared and stained with HE **(a)**, and the proportion of empty versus whole lacunae was calculated (**b**). Scale bars = 100 (upper) or 10 μm (lower panels). Data shows the mean percentage (%) of empty versus whole lacunae ± SD (n = 3, ***P < 0.001). Arrowheads indicate empty lacunae. Representative data of at least two independent experiments are shown.

Figure S2 Osteonecrosis formation occurs in mice administered anti-RANKL antibody.

Wild-type mice were administered alendronate (Ale) or anti-mouse RANKL antibody (anti-RANKL, 1mg/kg, twice a week) for two weeks. Then, infectious osteomyelitis was established by surgical injection of *Streptococcus aureus* into left femurs. Seven days after surgery, cortical bone sections were stained with HE **(a)**, and the proportion of empty versus whole lacunae was calculated (**b**). Bar = 100 (upper) or 10 μm (lower panels). Data shows the mean percentage (%) of empty versus whole lacunae ± SD (n = 3, NS, not significant.). Representative data of at least two independent experiments are shown.

Figure S3 Osteonecrosis development is not effectively blocked by teriparatide.

Wild-type mice were administered alendronate (Ale) for two weeks. Then infectious osteomyelitis was established in the left femurs as in Figure S2. PTH (80μg/kg) or PBS was injected subcutaneously at the time of surgery and subsequently twice a week for one week. Seven days after surgery, cortical bone sections were prepared and stained with HE **(a)**, and the proportion of empty versus whole lacunae was calculated (**b**). Scale bars = 100 (upper) or 10 μm (lower panels). Data shows the mean percentage (%) of empty versus whole lacunae ± SD (*n* = 3, NS, not significant). Representative data of at least two independent experiments are shown.

Figure S4 Teriparatide (PTH) administration promotes bone turnover.

Wild-type mice were administered PBS or alendronate for two weeks, and PTH was administered once infectious osteomyelitis was established surgically (PTH). Seven days after surgery**,** we prepared bone sections of the right femurs on the non-infected side and stained them with HE **(a)**. Comparable sections were stained with purified mouse anti-PCNA followed by Alexa488-conjugated goat anti-mouse Ig' (**b**). DAPI served as a nuclear stain. Scale bars = 100μm.

Figure S5 Osteonecrosis develops in the infectious but not the non-infectious (control) side.

Wild-type mice were administered alendronate for two weeks. Then, infectious

osteomyelitis was established by injection of *Streptococcus aureus* into the left femurs (infection). Right femurs served as controls (non-infection). Seven days later, cortical bone sections were prepared and stained with HE **(a)**, and the proportion of empty versus whole lacunae was calculated (**b**). Scale bars = 100 (upper) or 10 μm (lower panels). Data shows the mean percentage (%) of empty versus whole lacunae ± SD (n = 3, ***P < 0.001). Representative data of at least two independent experiments are shown.

Figure S6 Osteoclastogenesis is significantly inhibited while TNFα expression is significantly upregulated by treatment of osteoclast progenitors with a PG lysate. Wild-type mice were administered alendronate for two weeks. Then, infectious osteomyelitis was established by surgical injection of *Streptococcus aureus* (SA) or 1.8×10^7 CFU *Porphyromonas gingivalis* (PG) into the left femurs. Seven days after surgery, cortical bone sections were prepared and stained with HE **(a)**, and the proportion of empty versus whole lacunae was calculated (**b**). Scale bars = 100 (upper) or 10 μm (lower panels). Osteoclast progenitors were isolated from wild-type mice and cultured in the presence or absence of M-CSF (M) and RANKL (R) with or without PG lysate (**c, d**). Osteoclast formation was evaluated by TRAP staining (**c**) or realtime PCR analysis of expression of *Cathepsin K* **(***Ctsk)* or *NFATc1. TNFα* expression was also analyzed by realtime PCR (**d**). Data represent mean *Ctsk, NFATc1* or *TNFα* expression relative to *β-actin* ± SD (n = 3). **P < 0.01; ***P < 0.001; NS, not significant. Representative data of at least two independent experiments are shown.

Figure S7 TNFα is required for alendronate-induced osteonecrosis.

Wild-type (control) or TNFα-deficient (TNFα KO) mice were administered alendronate for two weeks. Infectious osteomyelitis was then established by *Streptococcus aureus* infection of left femurs of control or TNFα KO mice. One week later, cortical bone sections were prepared and stained with rabbit anti-single standard DNA (ssDNA) followed by Alexa488-conjugated goat anti-rabbit Ig'. DAPI was used as a nuclear stain. Scale bars = 100μm.

Figure S8 Expression of pro-inflammatory cytokines is significantly upregulated in femurs by infectious osteomyelitis.

Wild-type mice were injected with (+) or without (-) alendronate (Ale) for two weeks. Then, infectious osteomyelitis was established by surgical injection of *Streptococcus aureus* into the left femurs. Seven days later, mice were necropsied, and femurs were removed and analyzed for expression of *TNFα, IL-1β, IL-6, IL-17a* and *IL-17f* by realtime PCR. Data represent mean indicated gene expression relative to *β-actin* ± SD (n = 3). *$P < 0.05$; **$P < 0.01$; ***$P < 0.001$. Representative data of at least two independent experiments are shown.

Figure S9 Osteonecrosis induced by infectious osteomyelitis and alendronate. administration is significantly blocked in IL-1α/β- or IL-6-deficient mice.

Wild-type (control), IL-1α/β-deficient (**IL-1 DKO**), IL-6-deficient (**IL-6 KO**) mice, and IL-17A/F-deficient (**IL-17 KO**) mice were administered alendronate for two weeks. Then, infectious osteomyelitis was established by surgical injection of *Streptococcus aureus* into the left femurs. Cortical bone sections were prepared and stained with HE (**a**), and the proportion of empty versus whole lacunae was calculated (**b**). Data shows the mean percentage (%) of empty versus whole lacunae ± SD ($n = 4$, $^{**}P < 0.01$; NS, not significant). Scale bars = 100 (upper in **a**) or 10 μm (lower in **a**). Representative data of at least two independent experiments are shown.

Figure S10 1,25(OH)$_2$D$_3$ and ED71 significantly block osteonecrosis development in mice with infectious osteomyelitis treated with alendronate.

Wild-type mice were administered alendronate for two weeks. Then, infectious osteomyelitis was established by surgical injection of *Streptococcus aureus* into the left femurs. Vehicle (control), 1,25(OH)$_2$D$_3$ or ED71 was subcutaneously injected twice a week for two weeks before surgery and subsequently twice a week. Seven days after surgery, cortical bone sections were prepared and stained with HE **(a)**, and the proportion of empty versus whole lacunae was calculated (**b**). Scale bars = 100 (upper) or 10 μm (lower panels). Data shows the mean percentage (%) of empty versus whole lacunae ± SD ($n = 3$, $^{*}P < 0.05$; $^{**}P < 0.01$). Representative data of at least two independent experiments are shown.

Figure S11 Ectopic bone formation beneath the periosteum in the osteonecrosis model is inflammatory cytokine-dependent.

Wild-type or indicated knockout mice were administered vehicle (PBS, **a**) alendronate (Ale, **a-g**) or anti-RANKL antibody (**b**) for two weeks. Then, infectious osteomyelitis was established by surgical injection of *Streptococcus aureus* (SA, **a-g**) or *Porphyromonas gingivalis* (PG, **c**) into the left femurs. Some mice were administered etanercept or control (ISO), or PTH, ED71 or 1,25$(OH)_2D_3$ one week before surgery (etanercept or ISO), or when infectious osteomyelitis was established (PTH), or two weeks before surgery (ED71 or 1,25$(OH)_2D_3$). Seven days after surgery, cortical bone sections were prepared and stained with HE and the thickness of original cortical bone (solid double-headed arrow in panel a, right) or ectopic bone (blue dashed double-headed arrow in panel a, right) beneath the periosteum was measured. Scale bar = 100 μm. Data represents mean relative ectopic bone thickness relative to native cortical bone thickness (%) ± SD (n = 4, *P < 0.05). Representative data of at least two independent experiments are shown.

Figure S12 VD3 or ED71 does not inhibit inflammatory cytokine expression in macrophages or osteoclasts.

Bone marrow cells were isolated from wild-type mice and cultured in the presence or absence of M-CSF (M) (**a**) or M-CSF (M) and RANKL (R) (**b**) with or without either ED71 or 1,25$(OH)_2D_3$ (10^{-8}M, 10^{-7}M). Expression of *TNFα, IL-1β* or *IL-6* was analyzed by realtime PCR. Data represent mean indicated transcript levels relative to *β-actin* ± SD (n = 3). NS, not significant; *P < 0.05; **P < 0.01; ***P < 0.001. Representative data of at least two independent experiments are shown.

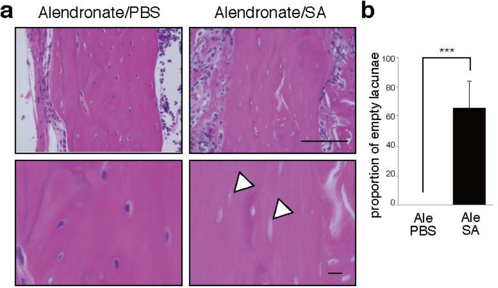

Figure S1 Morita M et al

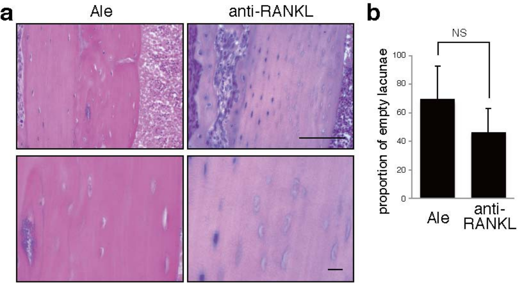

Figure S2 Morita M et al

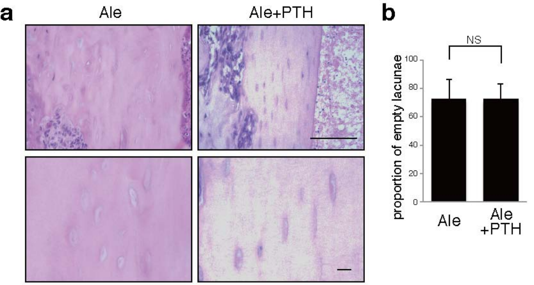

Figure S3 Morita M et al

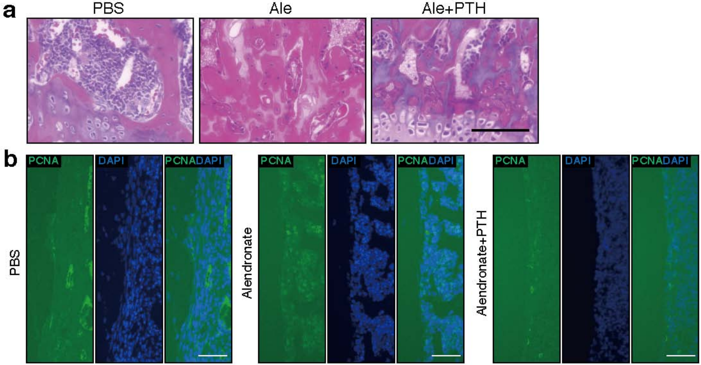

Figure S4 Morita M et al

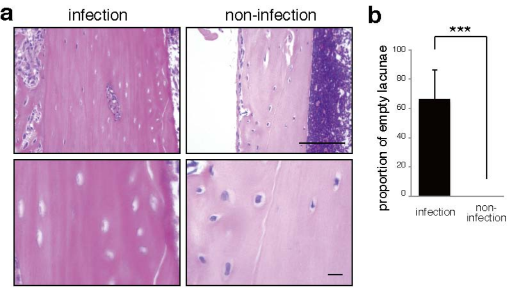

Figure S5 Morita M et al

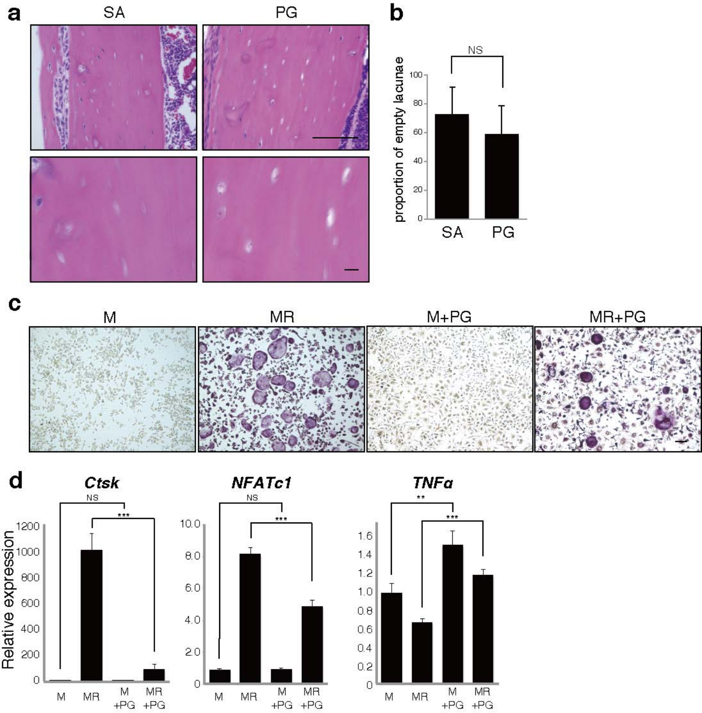

Figure S6 Morita M et al

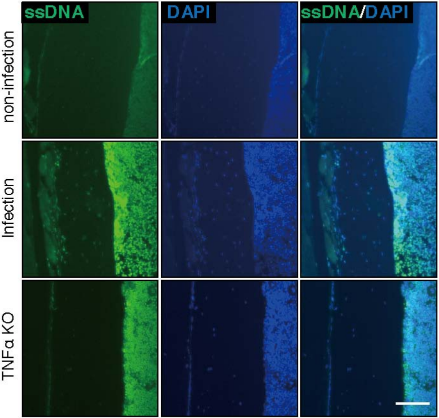

Figure S7 Morita M et al

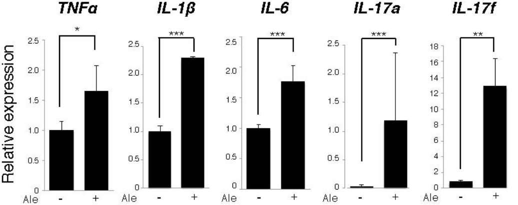

Figure S8 Morita M et al

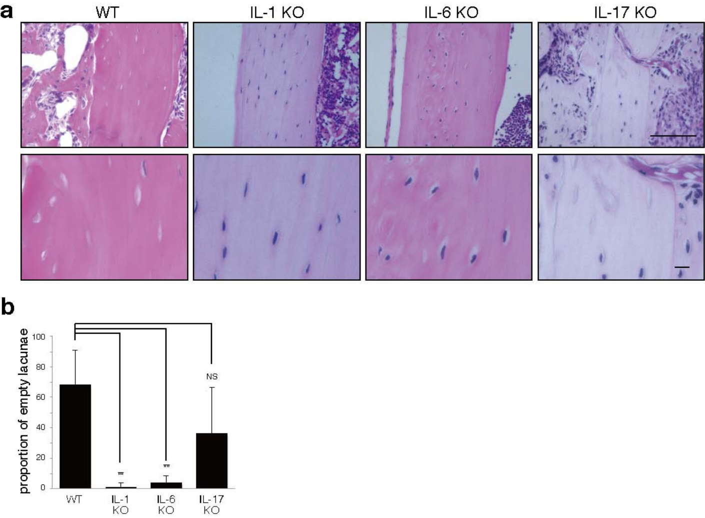

Figure S9 Morita M et al

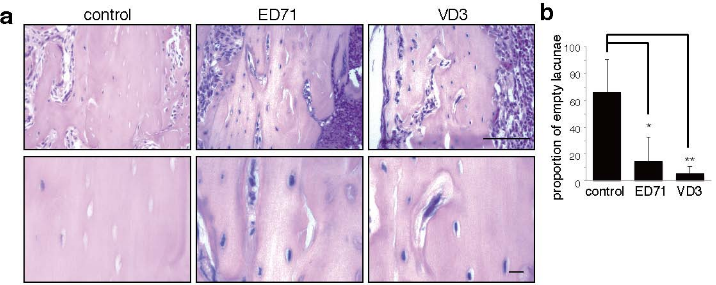

Figure S10 Morita M et al

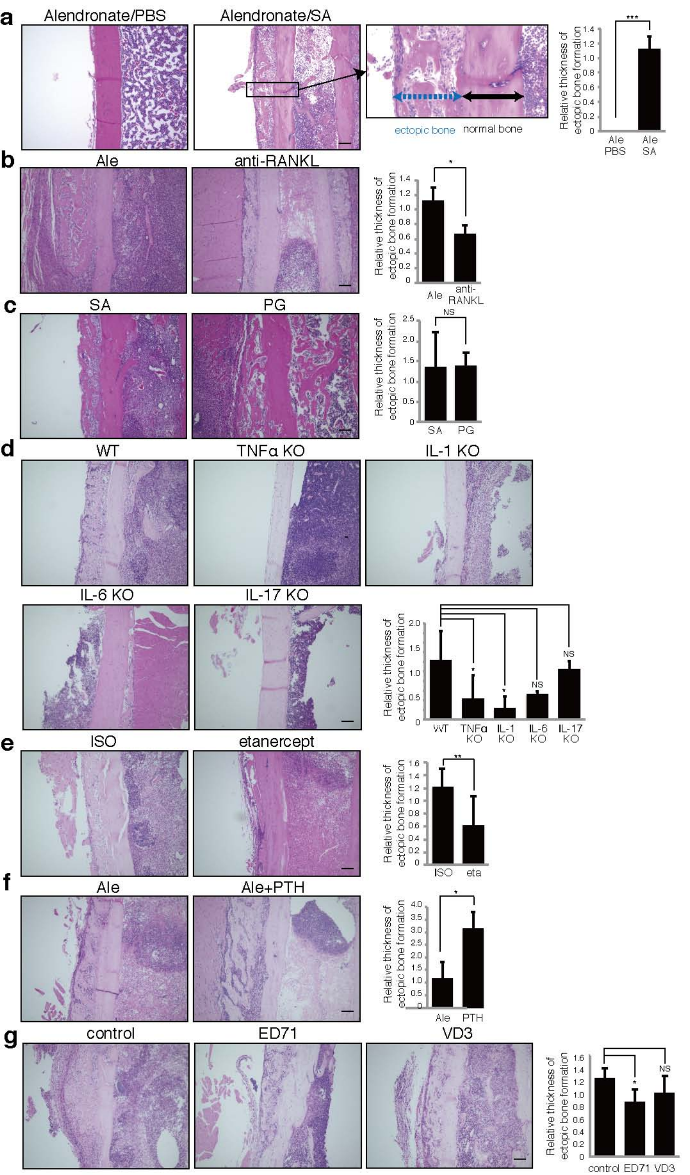

Figure S11 Morita M et al

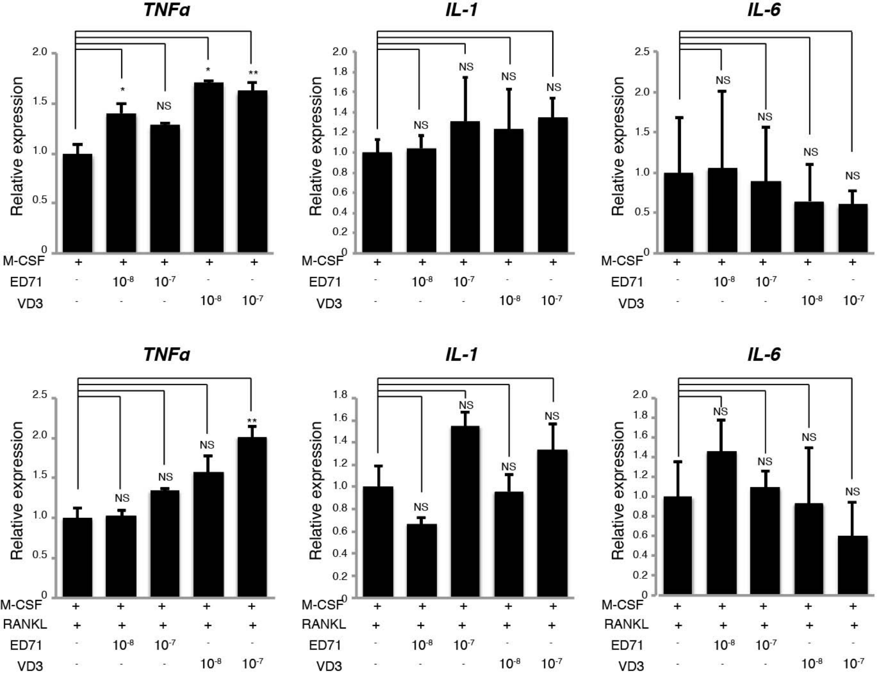

TNFa
IL-1
IL-6
Relative expression
M-CSF
RANKL
ED71
VD3
10-8
10-7
NS
*
**
